# Influence of Water Absorption on the Low Velocity Falling Weight Impact Damage Behaviour of Flax/Glass Reinforced Vinyl Ester Hybrid Composites

**DOI:** 10.3390/molecules25020278

**Published:** 2020-01-09

**Authors:** Angeline Paturel, Hom Nath Dhakal

**Affiliations:** Advanced Materials and Manufacturing (AMM) Research Group, School of Mechanical and Design Engineering, University of Portsmouth, Portsmouth PO1 3DJ, UK; Angeline.Paturel@myport.ac.uk

**Keywords:** flax fibres, impact damage, moisture absorption behaviour, biocomposites, delamination, μ-CT imaging

## Abstract

Due to rigorous new environmental legislations, automotive, marine, aerospace, and construction sectors have redirected their focus into using more recyclable, sustainable, and environmentally friendly lightweight materials driven by strengthening resource efficiency drive. In this study, the influence of moisture absorption on flax and flax/glass hybrid laminates is presented with the aim to investigating their low velocity impact behaviour. Three different types of composite laminates namely, flax fibre reinforced vinyl ester, flax fibre hybridised glass fibre and glass fibre reinforced vinyl ester composites were fabricated using resin infusion technique. The moisture immersion tests were undertaken by immersing the different specimens in sea water bath at room temperature and 70 °C at different time durations. The low velocity falling weight impact testing was performed at 25 Joules of incident energy level and impact damage behaviour was evaluated at both ageing conditions using scanning electron microscopy (SEM) and X-ray microcomputed tomography (micro CT). The percentage of moisture uptake was decreased for flax vinyl ester specimens with glass fibre hybridisation. The maximum percentage of weight gain for flax fibre, flax/glass hybrid and glass fibre reinforced composites immersed at room temperature for 696 h is recorded at 3.97%, 1.93%, and 0.431%, respectively. The hybrid composite exhibited higher load and energy when compared flax/vinyl ester composite without hybridisation, indicating the hybrid system as a valid strategy towards achieving improved structural performance of natural fibre composites. The moisture absorption behaviour of these composites at room was observed to follow Fickian behaviour.

## 1. Introduction

The ever-increasing demand for lower CO_2_ emission has driven intensive research into sustainable lightweight materials in transport sector including marine, automotive and aerospace applications [1,2,3]. Composite materials have progressed from traditional fibre composite materials, reinforced with synthetic fibres, such as glass or carbon fibres, to natural fibre composite materials. Some natural fibres allow mechanical reinforcements for impact, tensile and flexural properties, such as flax or hemp. These are stiff and lighter reinforcements (density 1.5 g/cm^3^) compared to glass fibres (density 2.5 g/cm^3^), having a better strength to weight ratio to glass fibres. This specific advantageous property favours the use of these lightweight reinforcements to manufacture sustainable composites suitable for transport sector [1,4]. Additionally, the natural fibres offer better environmental sustainability, recyclability, biodegradability and renewability [5,6].

Natural fibre reinforced composites with high strength and stiffness, together possessing acceptable mechanical properties closer to conventional glass fibre composites, are of great importance in terms of developing lightweight and environmentally sustainable composite materials for structural applications. Despite several benefits, due to their inferior mechanical properties in comparison to their conventional counterparts has limited the use of these composites mainly in non-structural and semi-structural applications, thus limiting large scale industrial uses [7]. Indeed, natural fibre reinforced composites applications are limited by their low stiffness and low impact properties, inadequate to secure a structural part, while traditional fibre reinforced composites perfectly meet the requirements for structural applications [8,9,10,11,12].

Hybrid fibre composite materials are a combination of more than one type of fibre in the same matrix, two types of fibre being the most beneficial. Thus, hybrid system is composed of elements of two separate systems. In recent years, in order to overcome the drawbacks, hybrid approach has been recommended as a viable means to tailor and enhance the overall mechanical and long-term durability of natural fibre composites and biocompostes. However, understanding hybrid materials and their compatibility (interfacial layer characteristics), failure behaviours, structure property relationships, and dealing with their natural variability, especially in the case of natural fibres as reinforcements are the most challenging issues for the use of natural fibre reinforced composites and biocomposites in transport sector [13,14].

The properties of the natural fibre hybridised with glass fibre composites have been investigated by several authors [15,16]. These reports have suggested that hybridisation of natural fibre with short glass fibre, could increase the storage modulus of biocomposites. Indeed, hybridising natural fibres with a stronger synthetic fibre can significantly improve the strength and stiffness of the natural fibre reinforced composites [17].

The understanding of moisture absorption behaviour and its influence on the mechanical properties is important for composite materials to be used in marine applications. Indeed, all polymer composites are susceptible to moisture absorption in humid atmosphere and when immersed in water. The effect of moisture absorption leads to the degradation of fibre-matrix interface region creating poor stress transfer efficiencies resulting in a reduction of physical, mechanical and dimensional properties [1,18]. Numerous reported works that have highlighted where hybridising natural fibres with glass and carbon fibre, has contributed significant improvements in mechanical, thermal and moisture repellence behaviour of natural fibre composites. For example, the work carried out by Jawaid et al. [19] investigated the addition of glass fibre to palm fibre and they reported that the moisture absorption behaviour of the composites was decreased. Similarly, the study conducted by Yongly et al. [9] highlighted that by hybridising flax fibres with glass fibres could allow to obtain a composite lighter in weight, higher strength and modulus and greener than synthetic materials. Some recent works focused on the impact of the water absorption on the mechanical properties of hybrid composites [20,21,22]. However, there are not many reported works on the influence of flax/glass fibre hybridisation on the moisture absorption behaviour at different temperatures and their effects on the mechanical properties of natural fibre composites.

The reported work in this study focuses on the development of lightweight flax/glass hybrid composites, investigating their moisture absorption behaviour at different temperatures and correlating the moisture absorption on the low velocity falling weight impact performance of flax and flax/glass hybrid vinyl ester biobased composites potential for transport applications. The findings of this study will help in understanding the impact damage behaviour and failure mechanisms of natural fibre hybrid composites towards using these composites for semi-structural and structural applications.

## 2. Results and Discussion

### 2.1. Moisture Absorption Behaviour

**Figure 1:**
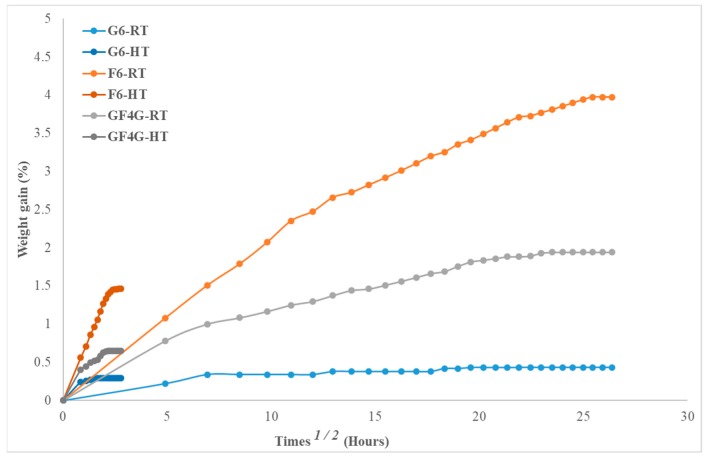
Moisture absorption versus square root of time at room temperature and high temperature.

The moisture absorption behaviour for three composite specimens studied are analysed by considering two important parameters namely the moisture uptake percentage and the diffusion coefficient. The moisture uptake and the diffusion coefficient results calculated using percentage moisture gain versus square root of time are presented in Table 1. The traces of moisture uptake for various samples are shown in Figure 1.

#### 2.1.1. Moisture Absorption Behaviour at Room Temperature

The maximum percentage of weight gain for glass fibre reinforced composites (G6), flax fibre reinforced composites (F6) and glass/flax fibre hybrid composites (GF4G) immersed at room temperature for 696 h is recorded at 0.431%, 3.972%, and 1.938%, respectively, as presented in Table 1. It can also be highlighted that the samples were fabricated with high quality with low void contents (about 4%) leading to a low sensitivity to temperature and water ingress.

The Figure 1 shows the water uptake behaviour for all specimens at room temperature and at high temperature (70 °C) For all specimens immersed at room temperature show moisture uptake linear in the beginning, then slows down and approaches to saturation after prolonged period of time, following a Fickian diffusion behaviour. It is worth noting that glass fibre reinforced composite samples exhibited low moisture gain and reached to saturation quite early compared to flax alone and flax/glass hybrid composites. It is evident from the results (Figure 1) that both the initial rate of water absorption and the maximum water uptake is highest for flax (F6) specimens followed by flax/glass hybrid specimens (GF4G) and lowest for glass fibre vinyl ester composite (G6).

This phenomenon can be explained by the hydrophilic nature of natural fibres such as flax. When a natural fibre reinforced composite is exposed to moisture, the fibres swell, leading to micro cracks of the resin. In the case of flax fibre, the high cellulose content which is about 70%, causes water ingress at higher rate into the interface through the micro cracks causing to more swelling of the fibres. Moreover, flax fibres contain many hydroxyl groups (-OH) in their structures. These groups form a large number of hydrogen bonds between the macromolecules of the cellulose and polymer. The presence of a large number of -OH group leads to a low moisture resistance, hence leading to a poor interfacial bonding between fibre and the matrix [23].

For the glass samples, glass fibres are not hydrophilic such as flax fibres so the water principally penetrates the matrix part, in the voids created during the manufacturing process but does not ingress so much into the fibre limiting the water ingress.

Regarding the hybrid composites, glass fibres are placed on the exterior of the samples which restrain the penetration of water. However, water still penetrates through the matrix voids and the sample sides, then it reaches the fibres, which diffuse the water by capillarity but with a slower rate compared to flax vinyl ester samples without hybridisation.

#### 2.1.2. Moisture Absorption Behaviour at High Temperature

The maximum percentage weight gain for G6, F6, and GF4G samples immersed at high temperature at 70 °C for about 8 h is recorded at 0.294%, 1.466%, and 0.647%, respectively, as presented in Table 1. However, these moisture gains for all samples at 70 °C are significantly lower than gains for samples immersed at room temperature. Thus, in this case, it is signified that the temperature does not increase the water absorption percentage. It can be envisaged that if these samples are immersed for a prolonged period of time, the second stage absorption may have occurred.

Yet, in the literature, several papers have highlighted the effect of temperature on water absorption behaviour: it often seems that the higher the temperature, the greater the moisture uptake. Dhakal et al. [1] have performed water absorption tests at RT and HT (100 °C) for hemp fibre reinforced composites. They proposed clear evidence that temperature increases water absorption with a high diffusion coefficient for high temperature environment compared to room temperature. This is probably attributed to a different diffusivity of water into the material, caused by thermal expansion due to the heat. Indeed, because of the heat, the interface becomes weaker, a debonding between fibre and matrix takes place, leading to an ease to flow for the water molecules, creating a good path making easy for water to transport at faster rate. This further promotes fibre swelling, leading to matrix cracks and fibre–matrix interface degradation at an accelerated rate.

Theoretically, the moisture diffusion in a composite is composed of complex mechanisms. The absorption of moisture by a composite, when this one is in humid atmosphere or when it is immersed in water, leads to the hydrophilic natural of fibre swelling, to the degradation of fibre–matrix interface region creating and so, to a reduction of mechanical and dimensional stability [24].

In a composite, three mechanisms involve the moisture diffusion. The first one is the diffusion of water molecules inside the micro gaps between polymer chains. About the second one, the capillary motion allows the transport of the water into the gaps and causes the flaws at the interfaces between fibre and the matrix, themselves caused by a poor wetting and a poor impregnation during the manufacturing stage. The last one is the transport of micro-cracks in the matrix arising from the swelling of fibres [18].

The high temperature used in this study which was 70 °C maybe was not high enough to make the matrix weak and to create good path to let the water to go into fibre matrix interfaces reaching the saturation point rapidly hence lower moisture uptake percentages.

Moreover, at high temperature, the moisture saturation time is significantly shortened, from 696h to 8hrs only. This shows that sorption at high temperature takes much less time to reach equilibrium than sorption at room temperature. This can be suggested that the high temperature moisture testing shortens the time and provides the results in a short time compared to room temperature moisture absorption tests.

Thus, slopes of moisture uptake traces as at high temperature as shown in Figure 1 are a lot steeper than those of immersed at room temperature. Similarly, the diffusion coefficient for high temperature samples are significantly higher than those of immersed at room temperature. This highlights a difference between the sorption behaviour at room temperature and high temperature as well as ageing mechanisms [25].

For the HT absorption, a non-Fickian behaviour can be considered. If the samples were stayed immersed a longer time, the temperature and/or the water would have damaged the matrix interface and a good path for the water would have been created. Thus, in this case, a two-stage absorption behaviour could have been realised.

#### 2.1.3. Diffusion Coefficient

Table 1 also presents the diffusion coefficients for both room temperature and high temperature- water immersed specimens. Diffusion coefficient refers to the measure of mobility of diffusing species in the material. It can be seen that the diffusion coefficient values increase with an increase in initial slopes of the traces, hence with an increase in temperature. This increase is noticeable for each specimen. Diffusion coefficient values of F6 is the highest, because its moisture content percentage is also the highest, followed by the hybrid system, then by the glass fibre composites. The diffusion coefficient values at RT are lower than at HT, probably because the increasing in temperature leads to a weaker interface increasing the water diffusion capability [26].

### 2.2. Effect of Moisture Absorption on the Low Velocity Impact Behaviour

Natural fibres are hydrophilic in nature and they absorb water from their environment. However, this water acts on the fibres as a plasticizing agent and as a swelling agent [27]. When the absorbed water diffuses into the fibres, this can lead to a poor interfacial strength in composites and to a significant decrease in the mechanical properties of the material.

To highlight the effect of moisture absorption on the low velocity impact characteristics, low velocity impact tests have been performed on dry G6, F6 and GF4G samples and on wet samples at room temperature and high temperature and the results are discussed in the following sections.

#### 2.2.1. Load Versus Deflection Traces

**Figure 2:**
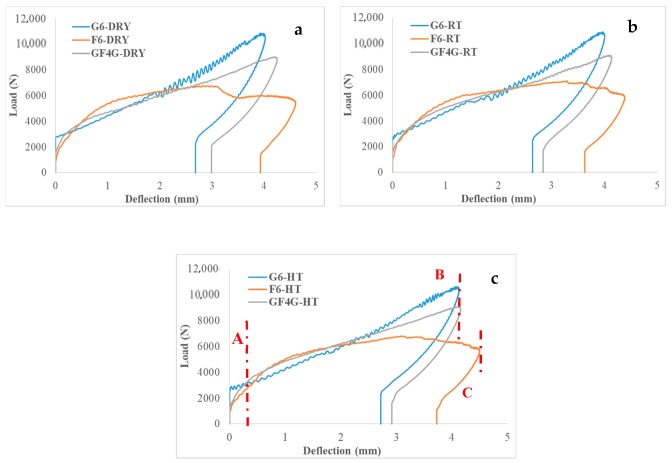
Load versus deflection traces for (**a**) dry samples, (**b**) room temperature wet samples and (**c**) high temperature wet samples.

Figure 2 shows the load–deflection behaviour for impacted G6, F6, and GF4G hybrid composite specimens. Table 2 presents the maximum load and the maximum deflection of dry samples compared to water immersed at RT and HT specimens. 

In the three conditions compared (dry, RT, and HT), the maximum deflection is quite similar. When compared to the maximum load, the glass fibre specimens exhibited the highest load followed by the flax/glass hybrid composites. It is worth noting that the load bearing capability of GF4G is near to that of G6, a small difference is noticeable, this is an evidence of the positive hybrid effect.

Then, it can be seen that the deflection is highest for F6, following by the hybrid composites GF4G and then G6. If the deflection is high, it means that the ductility is also high for that particular sample. With higher deflection, it leads to a better dissipation of energy (energy absorbed increasing). Compared to G6 samples, the hybridisation permits to increase this ductility, which is another positive effect of hybrid system. Thus, the diameter of the glass fibre is more uniform, leading to a load bear capability higher whereas flax fibre is more ductile leading to a high deflection. A trade-off between these two properties allows to notice the positive effect of hybridisation. The hybrid effect is the same in the three cases as it leads to an enhancement of the properties without deterioration.

#### 2.2.2. Load Versus Time Traces

**Figure 3:**
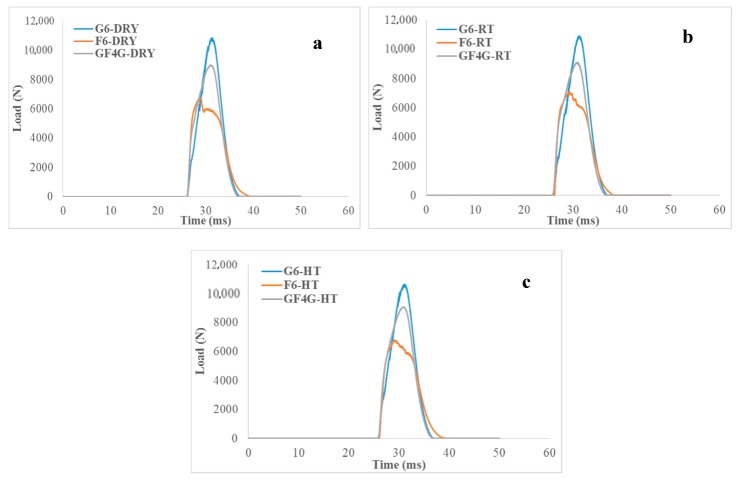
Load versus time traces for (**a**) dry samples, (**b**) room temperature wet samples and (**c**) high temperature wet samples.

Figure 3 shows the load–time behaviour for impacted glass, flax and hybrid composite specimen and Table 3 presents the difference between the maximum load and the maximum test time of dry samples compared to RT and HT wet samples. 

In this case, the observations about the load are the same than previously. Here, the test time comes into play. The time the striker was in contact with the impacted specimens is approximately the same for each composite (see Table 3). However, time taken is a bit longer for flax fibre composites, the curve takes slightly more time to go back to the initial value. This is an indication of flax fibre composite having better impact resistance behaviour, which signify that the toughness of flax fibres is higher than that of other composites and the same is true for the ductility.

Furthermore, two discrete peaks are noticeable for the flax composite samples in dry conditions while for RT and HT immersed flax samples, curves are smoother and show only one peak.

#### 2.2.3. Work Versus Time Traces

**Figure 4:**
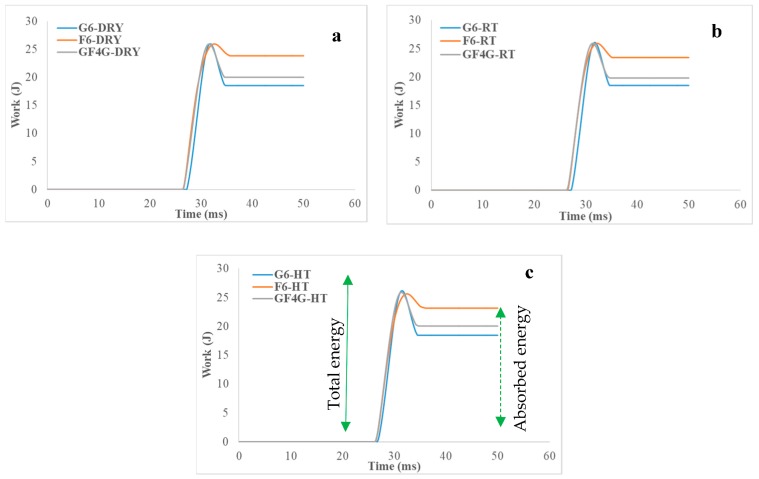
Work versus time traces for (**a**) dry samples, (**b**) room temperature wet samples and (**c**) high temperature wet samples.

Figure 4 shows the variations in energy absorbed versus the test time for the three composites studied and Table 4 presents the difference between the maximum work and the maximum time of dry samples, RT, and HT wet samples.

The peak load shows the maximum energy (given by the work (J)), which is also the total energy. Then, a drop is noticeable. The total energy less the drop value gives the absorbed energy quantity (Figure 4c). For all samples, work capacity is quite similar and is equalled more or less to 25 J.

In the three conditions, the energy absorbed by each sample types is quite similar. Nevertheless, an important difference between the samples is noticeable. Indeed, the F6 specimen absorbs almost all the energy, about 23.5 J, especially thanks to the ductility of its fibres and to hydroxyl group presence in fibres. The stronger the material, the less the energy absorption, so the glass fibre composite absorbs not much energy, about 18.5 J, because of the brittle nature of its fibres.

According to the hybrid composite, the absorption energy capability of GF4G sample is near than that of glass fibre specimen, the both are more or less the same, but the absorbed energy is about 20 J, being 7% higher for hybrid specimens, which presents further evidence of the positive hybrid effects.

### 2.3. Impact Damage Characteristics

#### 2.3.1. Visual Inspection of Specimens

As previously explained, specimens impacted with an incident energy of 25 J are not fully damaged, so they are non-penetrated.

**Figure 5:**
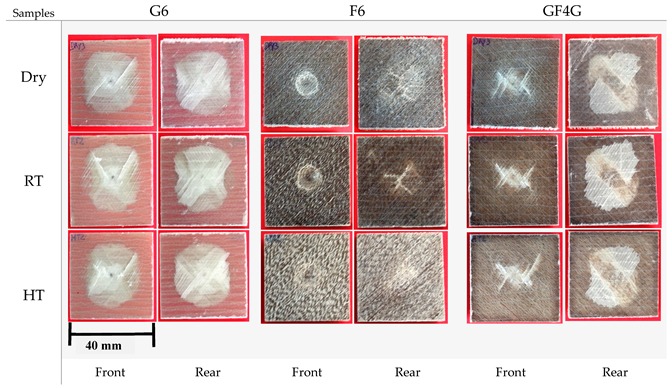
Pictures of dry, RT immersed and HT immersed glass fibre, flax fibre and hybrid specimen impacted at 25 J.

Figure 5 shows dry, RT, and HT conditioned samples after impact testing with an incident energy of 25 J. Before using the imaging tool, a visual inspection can be done.

For dry, RT and HT immersed G6 samples, an X-shaped damage is noticeable in the centre of the sample. It can be supposed that the energy is dissipated through the material following the X lines, where matrix cracking is visible. The damage area is quite large and is approximately the same for each condition.

Looking at the rear face, it can be supposed that the damage mode is the delamination because the different fibre plies and their orientations have appeared. Yet, delamination is a result of mismatch between the different fibre orientations and a delamination between plies at 0/90 °C is clearly observable. Moisture absorption does not seem to have an effect on the damage of glass fibre composites. This is probably due to the non-hydrophilic nature of glass fibres.

About flax fibre composites, the dry sample is damaged on one precise point, the damage is not extended. Thus, damage area is quite small. It highlights the high energy dissipation capability of flax fibres, due to their ductility behaviour. The rear face shows a quite large damage area with fibres possibly bended or broken, showing the energy dissipation.

According to the RT immersed sample, on the front face, the impacted area has less depth. On the rear face, the damage area is smaller, and the surface is flatter than in dry condition. Fibres bended are still observable but the look of the failure is quite different. Moisture absorption is sensed to increase the ductility; hence the energy dissipation seems to be higher. At HT, this phenomenon is most visible because the front face is slightly damaged and the presence of a bump on the rear face is noticeable but there are no important visible damages, highlighting more again the flax ductility. In this case, the temperature and/or the water ingress speed seem to increase the ductility of flax fibres.

For the hybrid system, front faces show a damage area resembling to a mix of the both others. The damage area is quite large but smaller than that of glass fibre samples and is spread from the impacted point to the sample sides. White lines appear, through which energy is probably dissipated. Rear faces resemble a lot of those of G6, matrix cracking and delamination between glass fibre ply and flax fibre ply, oriented at 0/90°, is clearly visible. Between dry and wet samples, there are no visible differences whilst HT immersed sample seems to be lightly less damaged.

#### 2.3.2. X-Ray Examination

**Figure 6:**
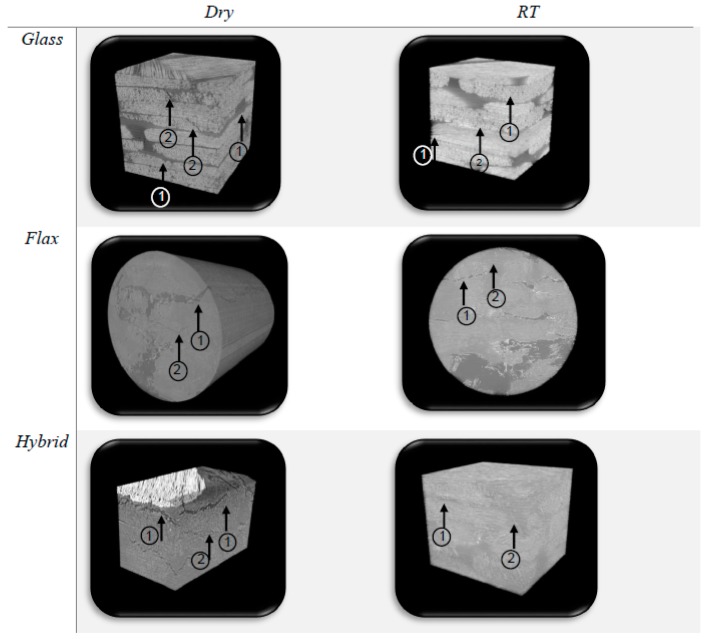
X-ray pictures of dry and RT immersed glass fibre, flax fibre and hybrid composites showing the damage mechanisms: (1) show delamination, (2) matrix cracking.

To complete the visual inspection, the X-ray examination allow to obtain more information about failure mechanisms. Indeed, the failure of dry and RT immersed samples are shown by Figure 6.

Two types of damage mechanisms are clearly noticeable: (1) the delamination, which occurs between two plies of different orientation and shows a breakage at the interface, and (2) the matrix damage, showing an incompatibility between the fibre and the matrix and leading to matrix cracking and/or to a debonding at the interface. There is no visible fibre breakage or bending.

In dry condition for glass fibre samples, the different layers are clearly visible, and an important number of matrix cracking and delamination damages are noticeable. The same is true for the RT immersed glass fibre samples because glass fibres are not sensitive to moisture absorption.

For flax fibre composites, the both damages are also noticeable. F6 sample seems to have more delamination damage than matrix cracking. Compare to glass fibre specimen, flax fibre sample has less of visible damages. For the RT immersed flax fibre samples, specimen seems to have less damages probably due to the high ductility of fibre.

Once again, the behaviour of the hybrid composites is between the both others but follows a trend near of that of flax composites. Indeed, some delamination zones are visible, but not much matrix cracking. In RT conditions, observations are the same.

X-ray scans confirm the visual observations. If the examination had been done for samples impacted with an incident energy of 50 J, as samples could have been fully damaged, fibre breakage, bending, or pull out would have probably been observed.

#### 2.3.3. SEM Characterisations

**Figure 7:**
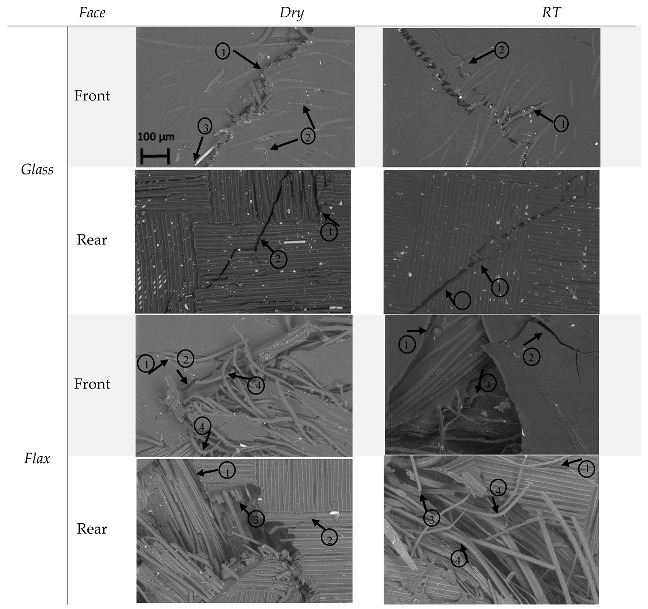

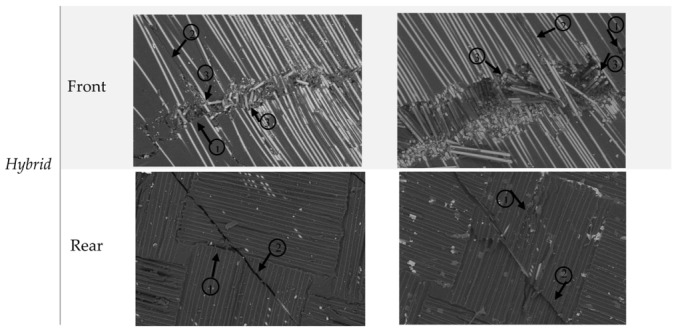
SEM pictures of dry and RT immersed glass fibre, flax fibre and hybrid composites with 300 × magnifications.

Then, to reach a high level of resolution, SEM characterisation gives more details about damage modes. The way of failing and the damages of dry and RT immersed samples are clearly shown by Figure 7. Characterisations have been done at a magnification of ×300 and the scale shown in the first image (front of the dry glass sample) is the same for the other images on the Figure 7.

The same damage modes are highlighted by SEM than by X-ray. However, in addition to matrix cracking and delamination, some fibre bending and fibre breakage are also clearly noticeable.

Indeed, for the dry and RT immersed glass fibre samples, (1) delamination and (2) matrix cracking are visible on the front face and also on the rear face. A few (3) fibre breakage are noticeable on the front face.

These both matrix cracking and delamination are also highlighted on the front face of flax fibre samples but there are also some (4) fibre bending, which the number is significant. It can be due to the high ductility behaviour of flax fibre, leading to an energy absorption higher. Furthermore, the number of fibre damages on RT immersed sample seems to be lowest than in dry sample. Indeed, the moisture uptake increased the ductility of the flax fibre, decreasing the impact on fibres. On the rear face, for the both dry and wet samples, fibre bending and fibre breakage can be seen. On the front face, matrix cracking, delamination and fibre breakage are highlighted on hybrid composite. The brittle aspect of glass fibre appears. On the rear face, there are no fibre damages.

The SEM characterisation confirm the visual inspection but, it also provides new information, such as fibre damages which are not visible to the naked eye or with the X-ray examination.

## 3. Experimental Procedures

### 3.1. Materials

In this study, the matrix material used was a vinyl ester resin because it possesses good properties and is not so expensive in comparison to epoxy matrix for example. It was mixed with 1% by weight of Methyl ethyl ketone peroxide (MEKP) catalyst for curing.

As reinforcement, a 0/90° oriented woven glass fibre and 0/90° oriented woven flax fibre, with a fabric weight of 300 g/m^2^ for the both, have been used. The typical chemical composition and mechanical properties of commonly used natural fibres are presented in Table 5.

### 3.2. Sample Fabrication

Firstly, flax fibres were dried at 100 °C for 24 h in an oven to remove storage moisture. Then, a combination of hand lay-up and vacuum infusion technique was used to prepare the laminated samples. Each sample is composed of 6 layers of fibre, either 6 glass fibre layers (G6), 6 flax fibre layers (F6), and 2 glass fibre layers and 4 flax fibre layers (GF4G). The vacuum infusion process is a technique that uses vacuum pressure to drive and impregnate resin into a laminate. Reinforcement and components destined to the process are put into the mould and the vacuum is applied. Once a complete vacuum is achieved, resin is literally sucked into the laminate via a pipe.

### 3.3. Moisture Absorption Test

The water absorption behaviour on flax fibre, glass fibre and hybrid glass/flax fibre reinforced unsaturated composites were investigated in accordance with BS EN ISO 62:1999.

The samples for water immersion testing were cut to a size of 60 × 60 × 3 mm^3^, 60 × 60 × 7 mm^3^ and 60 × 60 × 5.5 mm^3^ for glass fibre, flax fibre and glass/flax fibre hybrid specimens respectively.

Firstly, all the specimens have been placed in a desiccator at room temperature for 6 days before weighing them to the nearest 0.01 g to know their dehydrated weight. Water absorption tests were performed by immersing the specimens in a sea water bath, previously filtered with a 500 µm aperture, at room temperature for a long period to reach equilibrium.

Every day, at the same time, the specimens were taken out from the water and dried with a tissue to remove the surface water. The specimens were reweighed to the nearest 0.01 g within 1 min, in order to avoid the error due to evaporation, before removing them in the water.

The specimens were weighed every 24 h, up to 504 h exposure.

The same have been done at high temperature to determine water absorption at a higher temperature. Samples were immersed in filtered seawater in an environmental chamber with a constant temperature of 70 °C. By the same way that previously, the specimens were removed from the hot water (with a tong and dived in a cold bath for a few seconds), dried and weighted to the nearest 0.01 g at intervals of 30 min up to 8 h of exposure until the water saturation.

Then, Equation (1) allows calculating the percentage of water absorption in the polymer composites, by weight difference between the samples immersed in water and the dry samples [26].

(1) $$M_{t}=\frac{W_{t}{-W}_{0}}{W_{0}}\times 100$$

where M_t_ is moisture uptake, while W_0_ and W are the mass of the specimen before and during ageing, respectively.

The moisture content versus square root of time was plotted.

From the previous results, the rate at which water moves from the surface to the interior of the specimens is described by water diffusion coefficient. Diffusion coefficient D_x_, an important parameter in Fick’s law to determine the rate of absorption, can be calculated by using Equation (2), for the initial stage of the diffusion process [26];

(2) Dx=π (Mtt × h4 × Mt)2

where $M_{t}$ the maximum water uptake of the sample (%), h the thickness of the samples (m), and $\sqrt{t}$ the time square root (s).

### 3.4. Low Velocity Falling Weight Impact Testing

The low velocity instrumented falling weight impact tests were performed at room temperature on Zwick/Roell HIT230F (Stourbridge, UK) drop weight test machine. This one possesses an impactor of which the mass weight is 23.11 kg and is dropped from a height of 110 mm. In this case, the impactor is hemispherical with a diameter of 19.8 mm. The drop height generated 25 Joules of incident impact energy sufficiently enough to damage the samples without penetrate them. The load, the energy absorbed, and the test time were recorded by the machine. The test was conducted in accordance with the British Standard BSEN ISO 6603-2 recommendations [30].

### 3.5. Impact Damage Characterisation

#### 3.5.1. Scanning Electron Microscopy (SEM) Characterisation

The scanning electron microscope (SEM) allows performing morphological analysis of the composite. It specially allows to know the microstructure and the surface aspect of the samples but also to observe the impacted surface after testing to highlight the damage mechanisms. Thus, in order to perform morphological analysis of the samples, and to understand the effect of water absorption on the microstructure, the impacted surface of dried specimens and room temperature immersed specimens were examined using a scanning electron microscope (SEM) Zeiss EVO LS10 (Jena, Germany). Before examination, the samples have been placed in a desiccator to remove all the water of the samples, to avoid the evaporation during the characterisation. The specimens were also surface prepared, and the damaged area was imaged.

#### 3.5.2. X-Ray Micro CT Characterisation

Originally employed in medical imaging to obtain non-invasive images of human body, X-ray micro-computed tomography (micro-CT) is a useful characterisation tool in the field of materials, providing three-dimensional images, thanks to a high resolution from the micrometre to the sub-micrometre, which helps in understanding of relationship between structure, defects and ultimate failure. The impacted samples were imaged using micro-CT (micro-Computed Tomography) on a Zeiss Xradia 520 Versa system. The micro-CT scanner (Jena, Germany) was set to a voltage of 80 kV (60 kV for flax only specimens) and a power of 6 W (4 W for flax only specimens), while a source filter was used during the imaging process (Zeiss LE1 filter) and a flat panel detector.

## 4. Conclusions

The influence of moisture absorption at two different temperatures on the low velocity impact damage behaviour of flax and flax/glass hybrid laminates was investigated. From the experimental results, some conclusions can be drawn. Firstly, for an immersion at room temperature, it clearly appears that the flax fibre composite absorb much more moisture, leading to a higher diffusion coefficient. This phenomenon is due to the hydrophilic nature of natural fibres. In contrast, the glass composite possesses a very low moisture absorption percentage, and hence a low diffusion coefficient. The same observations are true for the high temperature immersion, the flax fibre composite absorbs the most of water, followed closely by hybrid system, then by glass fibre composites.

The damage characterisation on the low velocity impacted specimens, following by X-ray micro CT examination and SEM characterisations performed at both dry and water immersed conditions, highlight the potential of the hybrid system for structural applications as evidenced of high impact damage properties. Indeed, hybridised flax fibres with glass fibres significantly enhances the impact properties of the flax fibre reinforced composites. With a glass fibre layer on the top and another one on the rear, the load bearing capability has been increased by 25% compared to flax fibre composite alone and the energy absorption capability has increased approximately by 9% compared to glass fibre composite alone exhibiting improved impact damage characteristics by creating a balanced effect through the hybridisation of glass fibre onto flax fibre composite.

Finally, X-ray micro CT and SEM characterisations allowed for the understanding of the damage mechanisms of each composite. In the three cases, delamination and matrix cracking are visible and for flax fibre composite, fibre bending are noticeable because of its high ductility. For the glass fibre and the hybrid samples, fibre breakage is visible because of the brittle nature of glass fibres.

## Figures and Tables

**Table 1:** Moisture uptake and diffusion coefficient results.

	Room Temperature (23 °C)			High Temperature (70 °C)		
Different Samples	Moisture Absorption (%)	Diffusion Coefficient (m^2^/s)	Initial Slope of Plot (k) M(t) Versus t^1/2^	Moisture Absorption (%)	Diffusion Coefficient (m^2^/s)	Initial Slope of Plot (k) M(t) Versus t^1/2^
G6	0.43	2.5 × 10^−9^	0.01	0.29	2.30 × 10^−7^	0.08
F6	3.97	1.4 × 10^−8^	0.14	1.47	1.25 × 10^−6^	0.54
GF4G	1.94	8.5 × 10^−9^	0.07	0.65	7.70 × 10^−7^	0.20

**Table 2:** Maximum load and maximum deflection of dry samples and two condition wet samples.

Samples	Dry Condition		Room Temperature-Immersed Condition		High Temperature-Immersed Condition	
	Load (N)	Deflection (mm)	Load (N)	Deflection (mm)	Load (N)	Deflection (mm)
G6	10,866.00	2.60	10,928.00	2.60	10,656.00	2.70
F6	6769.00	3.90	7114.00	3.60	6814.00	3.70
GF4G	9005.00	3.00	9111.00	2.80	9083.00	2.90

**Table 3:** Maximum load and maximum time of dry samples, RT, and HT wet samples.

Samples	Dry Condition		Room Temperature-Immersed Condition		High Temperature-Immersed Condition	
	Load (N)	Time * (ms)	Load (N)	Time * (ms)	Load (N)	Time * (ms)
G6	10,866.00	11.00	10,928.00	10.90	10,656.00	10.90
F6	6769.00	12.10	7114.00	12.10	6814.00	12.30
GF4G	9005.00	10.40	9111.00	10.70	9083.00	10.50

* Time the striker was in contact with the specimens.

**Table 4:** Maximum work and maximum time of dry samples, RT and HT wet samples.

Samples	Dry Condition		Room Temperature-Immersed Condition		High Temperature-Immersed Condition	
	Work (J)	Absorbed Energy (J)	Work (J)	Absorbed Energy (J)	Work (J)	Absorbed Energy (J)
G6	25.90	18.50	26.10	18.50	26.20	18.50
F6	25.90	23.80	25.90	23.40	25.60	23.20
GF4G	25.90	20.00	25.90	19.80	25.90	20.10

**Table 5:** Chemical composition, physical and mechanical properties of commonly used natural bast fibres [28,29].

Fibres	Cellulose	Hemicelluloses	Lignin	Pectin	Density (g/cm^3^)	Moisture Gain (%)	Tensile Strength (MPa)	Young’s Modulus (GPa)	Failure Strain (%)
Flax	71	16.5	2.5	0.9	1.5	12	700	60	2.3
Hemp	81	20	4	0.9	1.5	12	530	45	3
Jute	67	16	9	0.2	1.4	17	325	38	2.5
E-Glass *	-	-	-	-	2.6	-	2000	76	2.6

* For comparison purpose.
